# Mass incarceration and the impact of prison release on HIV diagnoses in the US South

**DOI:** 10.1371/journal.pone.0198258

**Published:** 2018-06-11

**Authors:** Bisola O. Ojikutu, Sumeeta Srinivasan, Laura M. Bogart, S. V. Subramanian, Kenneth H. Mayer

**Affiliations:** 1 Brigham and Women’s Hospital, Boston, Massachusetts, United States of America; 2 Tufts University, Boston, Massachusetts, United States of America; 3 RAND Corporation, Santa Monica, California, United States of America; 4 Harvard School of Public Health, Boston, Massachusetts, United States of America; 5 The Fenway Institute, Boston, Massachusetts, United States of America; University of Queensland, AUSTRALIA

## Abstract

**Background:**

The purpose of this study was to determine the impact of prison release on HIV incidence in the southern region of the United States, the region with the highest rates of both incarceration and new HIV diagnoses nationwide.

**Methods:**

5-year HIV diagnoses rates were calculated at the ZIP code level for nine cities and metropolitan statistical areas in the US South (ZIP codes, N = 600). Multilevel regression models were constructed and adjusted rate ratios (ARRs) were estimated for overall, male and female HIV diagnoses rates.

**Results:**

Across the nine cities, in multilevel, multivariate analysis, controlling for income inequality (GINI coefficient), percent living in poverty and percent Non-Hispanic Black population, the ZIP code level overall HIV diagnosis rate was significantly associated with prison release [ARR 1.004 (95%CI 1.0007, 1.006), p<0.01]. A 10-person increase in prison release rate would result in a 4% increase in overall 5-year HIV diagnosis rate—approximately 9.4 additional cases per 100,000 population. In gender-stratified models, prison release rate was significantly associated with the ZIP code level HIV diagnosis rate for males [ARR 1.004 (95%CI 1.0004, 1.007), p<0.01], but not for females.

**Conclusions:**

In the southern region of the US, prison release is significantly associated with HIV incidence. HIV prevention interventions should promote timely linkage to ongoing treatment for released inmates living with HIV.

## Introduction

The United States is home to the largest imprisoned population in the world [1]. In 2014, more than 2.2 million individuals or 1 in 100 adults were incarcerated [2]. The epicenter of the epidemic of mass incarceration is the southern region of the US. Five southern states—Louisiana, Oklahoma, Arkansas, Alabama and Mississippi—lead the nation in imprisonment [3]. Comparing regional estimates, the rate of imprisonment (the number of prisoners sentenced to more than 1 year per 100,000 residents) in the South is more than twice that in the Northeast [3].

In the South, as is the case nationwide, mass incarceration disproportionately impacts black individuals. While approximately 40% of all incarcerated men are black, only 12% of males in the US are black [2,4]. More than 55% of the black population in the US lives in the South [5]. Therefore, the racial disparity (the ratio of incarcerated individuals who are black to the total black population) is less striking in the South. However, the absolute numbers and the prevalence of incarceration among black people is profound. In Louisiana, black individuals comprise 32% of the total population, and approximately 60% of incarcerated individuals [6].

Mass incarceration and the persistent racial disparities noted among imprisoned populations are a byproduct of the “War on Drugs”. Beginning in the 1970s, drug law violations were met with increasingly punitive policies, such as mandatory minimum sentencing for possession of drugs and differential sentencing for powder versus crack cocaine [7]. Rates of arrest, conviction and imprisonment of drug users in low-income, urban areas (where the war was largely staged) multiplied. As a result, the casualties of the war are disproportionately black and overwhelmingly poor. In the South, policies such as habitual offender (“three strikes”) laws for minor drug offenses, extended mandatory minimum sentences, and limited economic opportunities leading to recidivism continue to drive high incarceration rates [8].

**Fig 1:**
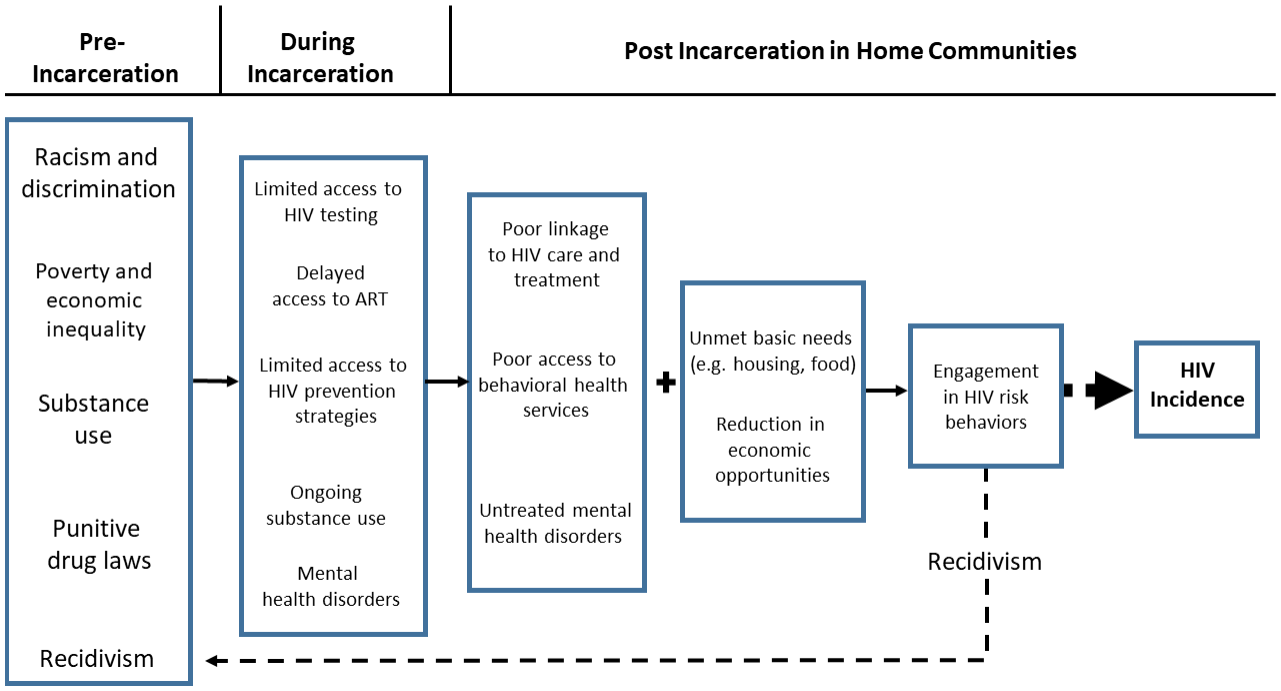
Drivers of onward HIV transmission from pre-incarceration to release in home communities.

Coincident with the rise of drug convictions has been the spread of HIV, particularly in the South where the highest number of new cases of HIV were noted in 2015 [9]. Elevated HIV transmission risk secondary to drug use has led to a significant number of people living with HIV (PLWH) who cycle in and out of prison [10,11]. In 2010, the prevalence of HIV infection amongst the incarcerated was approximately 5 times greater than among the non-incarcerated, largely due to engagement in high risk behaviors pre-incarceration [12]. Though incarcerated populations are at higher risk for HIV, few correctional facilities have implemented HIV testing, prevention and treatment services for inmates [13]. In addition, services critical to the comprehensive care of PLWH, such as behavioral health services to address substance abuse and treatment for mental health disorders are often suboptimal behind bars [14]. The lack of these services leads to challenges while incarcerated and post-discharge. Approximately 10 million individuals are released from prisons and jails back to their home communities annually [15,16]. Yet, most correctional facilities have not successfully implemented appropriate discharge planning for inmates [13]. Inmates living with HIV are often not linked to care and treatment, behavioral or mental health services in their home communities. Moreover, recently released PLWH, as well as other inmates, are at high risk for engagement in behavioral risk-taking behavior (e.g. transactional sex and drug use) when faced with unmet basic needs such as food and housing. Ensuring that services are in place at the time of discharge from correctional facilities to home communities may reduce the risk of onward HIV transmission. (Fig 1)

The purpose of this study was to determine the population-level association of prison release and HIV diagnoses in the South, controlling for known ecological associations (poverty, economic inequality, and race/ethnicity) [17,18]. Previous studies have focused on individual-level clinical outcomes. We hypothesized that the impact of prison release spans beyond the individual to the community.

## Methodology

### Level of analysis

All variables were obtained at the level of the ZIP code or ZIP Code Tabulation Area (ZCTA) which was the lowest level available for the outcomes data (5-year cumulative overall, male, and female HIV diagnoses) and prison release rates. ZIP codes are a system of postal codes devised by the United States Postal Service for the purpose of mail delivery. ZIP Code Tabulation Areas (ZCTAs) are calculated by the United States Census Bureau and approximate ZIP codes [19].

### Outcome

The primary outcomes for this study were 5-year cumulative overall, male, and female HIV diagnoses (over age 14) per 100,000 population (2010–2014) per ZIP code from 9 cities and metropolitan statistical areas (MSAs) in the US South: New Orleans, LA; Baton Rouge, LA; Miami, FL; Atlanta, GA; Houston, TX; Jacksonville, FL; Orlando, FL; Tampa, FL; and Columbia, SC. Cities were selected based upon the availability of data on HIV cases and prison release data. We obtained cumulative HIV case counts from www.aidsvu.org [20]. AIDSVu obtained release agreements with health departments in each city to allow for public access to ZIP code level data. ZIP-code-level HIV surveillance data reflect the reported residence at diagnosis for persons living with an HIV or AIDS diagnosis in the defined geographic area as of December 31, 2012. HIV diagnoses data reflect persons newly diagnosed with an HIV infection or infection ever classified as stage 3 (AIDS) between January 1, 2010 and December 31, 2014. Aggregate data (5 years) were combined due to small case counts in individual years. Denominators used to calculate diagnoses rates for ZIP Codes were obtained from the U.S. Census Bureau’s 2010 census ZCTAs.

### Prison release data

ZIP code linked data on prison release to the selected cities in 2008 were obtained from the Justice Mapping Center (www.justiceatlas.org) [21]. Data were obtained by the Justice Mapping Center from the Department of Corrections in each of the 9 cities. Address-level data were obtained from inmates prior to discharge and aggregated to the ZIP code level. Prison release rates were expressed per 1000 adult population. Data from 2008 were used to allow time between HIV transmission, diagnosis and case reporting.

### Covariates

To account for potential confounding we included independent variables that have been associated with HIV incidence and prevalence on a population-level in previous studies [17,18]. These include economic factors such as percentage of residents living below the federal poverty level, and income inequality as well as demographic factors such as percentage of Non-Hispanic Black residents. Income inequality was measured using the GINI coefficient, which varies between 0, which reflects complete equality and 1, which indicates complete inequality (i.e., one person has all the income and all others have none) [22]. All variables were obtained from the US Census Bureau’s American Community Survey 5-Year Estimates [23].

### Data analysis

Descriptive statistical analysis was conducted to examine the distribution of the data followed by bivariate correlations between dependent and independent variables using Spearman correlations. We also calculated median ZIP Code-level HIV diagnosis rates overall, male and female by low, medium, and high prison release rate (as defined by IQR) and presented them in box plots. Low was defined as < 1.1 per 1000 population (lowest 25% prison release rates), medium 1.1 to <5.0 per 1000 population (middle 50% prison release rates), and high greater than > = 5.0 per 1000 population (highest 25% prison release rates).

Next, we constructed multilevel models which recognize the existence of data hierarchies by allowing for residual components at each level in the hierarchy [24,25]. We used a random intercept multilevel model with two levels, ZIP codes and cities. The two-level model predicts HIV diagnosis rates as outcomes with the independent variables prison release rate, the percentage of population that is Black, percentage of population living below poverty and GINI coefficient with intercepts at both the city level and ZIP code level. Thus, the residual variance is partitioned into a between-location component (the variance of the city-level residuals) and a within-location component (the variance of the ZIP code-level residuals). We use a negative binomial model since the outcome is a count (HIV diagnoses over 5 years) offset by the population over the age of 14 in the ZIP code. Univariate regression models were constructed and expressed as unadjusted rate ratios. We predicted separate multivariate, multilevel models for the outcomes: overall, male and female HIV diagnosis rate for the 600 ZIP codes as adjusted rate ratios.

Based upon HIV surveillance data and differences between male and female HIV prevalence, we hypothesized that gender would modify the association of prison release rate with overall HIV diagnosis rates [26]. Therefore, in our second multilevel model we included an interaction term representing prison release and gender to test for effect modification. The gender effect modification model combines the ZIP code level diagnosis rates for both genders (1200 ZIP codes) and includes an interaction term which is 1 when the diagnosis rate is for males multiplied by the prison release rate. The other independent variables remain the same (percentage living in poverty, percentage black and GINI coefficient). Finally, we constructed models stratified by gender (male and female HIV diagnosis rates). Statistical hypotheses were tested using a p-value of 0.05. These models were estimated in R using the package glmmADMB.

## Results

### Univariate analysis

A total of 9 cities and 600 ZIP codes were included in this analysis. Median 5-year HIV diagnosis rate across all 9 cities was 156.6 (IQR 205.3) per 100,000 and ranged from a median of 91.3 (IQR 93.6) per 100,000 in Tampa, FL to 252.6 (IQR 326.7) per 100,000 in Baton Rouge, LA. The highest male 5-year HIV diagnosis rate 400.7 (IQR 421.2) per 100,000 was noted in Miami, FL. The highest female 5-year HIV diagnosis rate was 251.4 (IQR 303.4) per 100,000 in Baton Rouge, LA. The median prison release rate per 1,000 population across all cities was 2.3 (IQR 3.9). Median income inequality measured by the GINI coefficient was 0.4 (IQR 0.1), similar to data calculated for the entire US [22]. The median percent Non-Hispanic Black population was 2.9 (IQR 7.9). The median percent population living in poverty was similar to national estimates (14.9% vs 14.8%) [27] (Table 1).

**Table 1:** Overall, male and female 5-year HIV diagnoses rates and independent variables by city.

	Number of ZIP codes	Overall 5-year HIV Diagnosis Rate Median (IQR)	Male 5-year HIV Diagnosis Rate Median (IQR)	Female 5-year Diagnosis Rate Median (IQR)	Median Prison Release Rate (IQR)	Median GINI Coefficient (IQR)	Median Percent Non-Hispanic Black Population (IQR)	Median Percent Living in Poverty (IQR)
Total	600	156.6 (205.3)	262.4 (341.9)	98.6 (126.4)	2.3 (3.9)	0.4 (0.1)	2.9 (7.9)	14.9 (12.3)
Atlanta	117	189.2 (298.5)	329.7 (514.3)	89.9 (95.5)	2.6 (2.4)	0.44 (0.08)	7.1 (11.3)	16.9 (12)
Baton Rouge	17	252.6 (326.7)	364.3 (388.9)	251.4 (303.4)	4.4 (3.7)	0.44 (0.09)	11.4 (14.5)	20.1 (8.8)
Columbia	22	148.3 (117.2)	240.4 (177.2)	83.9 (58.5)	3.3 (3.7)	0.43 (0.06)	6.4 (6.4)	16.1 (11.9)
Houston	132	174.1 (193.8)	277.2 (280.3)	99.7 (113.2)	6.5 (4.8)	0.44 (0.08)	2.9 (4.8)	18.3 (16.5)
Jacksonville	36	120.6 (174.5)	233.0 (268.0)	130.8 (146.2)	4.7 (2.7)	0.44 (0.08)	3.4 (9.9)	16.2 (10.6)
Miami	73	223.5 (324.7)	400.7 (421.2)	108.2 (283.8)	4.6 (1.7)	0.47 (0.08)	1.4 (7.1)	19.4 (12)
New Orleans	31	238.3 (312.2)	330.7 (459.8)	153.9 (155.6)	27.7 (37.9)	0.49 (0.08)	13.5 (17.7)	23.3 (15)
Orlando	71	137.6 (111.6)	239.5 (205.6)	69.4 (49.5)	4.2 (1.9)	0.42 (0.06)	2.1 (2.7)	14.2 (7.8)
Tampa	101	91.3 (93.6)	152.9 (170.6)	85.7 (118.1)	3.7 (2.8)	0.44 (0.05)	1.6 (2.8)	15.1 (9.8)

### Bivariate analysis

In bivariate analysis (Spearman correlations), overall 5-year HIV diagnosis rate was moderately associated with prison release rate (r = 0.43, p<0.01). Stratifying the data by gender, we found that prison release rate was moderately associated with female (r = 0.47, p<0.01) and less strongly associated with male (r = 0.35, p<0.01) HIV diagnosis rate. Prison release rate was also moderately associated with percent population living in poverty (r = 0.57, p<0.01) and percent Non-Hispanic Black population (r = 0.47, p<0.01). (Table 2)

**Table 2:** Spearman correlations among independent covariates and overall, male, and female 5-year HIV diagnoses rates.

	Overall 5-year HIV Diagnosis Rate	Male 5-year HIV Diagnosis Rate	Female 5-year HIV Diagnosis Rate	Prison Release Rate	GINI Coefficient	Percent Living in Poverty
Male HIV Diagnosis Rate	0.97**					
Female HIV Diagnosis Rate	0.72**	0.65**				
Prison Release Rate	0.43**	0.35**	0.47**			
GINI Index	0.39**	0.43**	0.20**	-0.01		
Percent Living in Poverty	0.66**	0.61**	0.69**	0.57**	0.30**	
Percent Non-Hispanic Black Population	0.67**	0.61**	0.74**	0.47**	0.20**	0.68**

*p<0.05,

**p<0.01

By city, prison release rate was strongly associated with overall, male and female HIV diagnosis rate for 8 out of 9 locations included in this analysis. The strongest correlation was noted in Baton Rouge, LA (r = 0.88, p<0.01), followed by Jacksonville, FL (r = 0.67, p<0.01), New Orleans, LA (r = 0.64, p<0.01), Miami, FL (r = 0.59, p<0.01), Houston, TX (r = 0.58, p<0.01), Atlanta, GA (r = 0.46, p<0.01), Tampa (r = 0.41, p<0.01), and Orlando, FL (r = 0.40, p<0.01). Prison release rate was not associated with overall, male or female HIV diagnosis rates in Columbia, SC. For both male and female HIV diagnosis rates, the association with prison release rate was strongest in Baton Rouge, LA (r = 0.70 for males and r = 0.80 for females, p<0.01).

**Fig 2:**
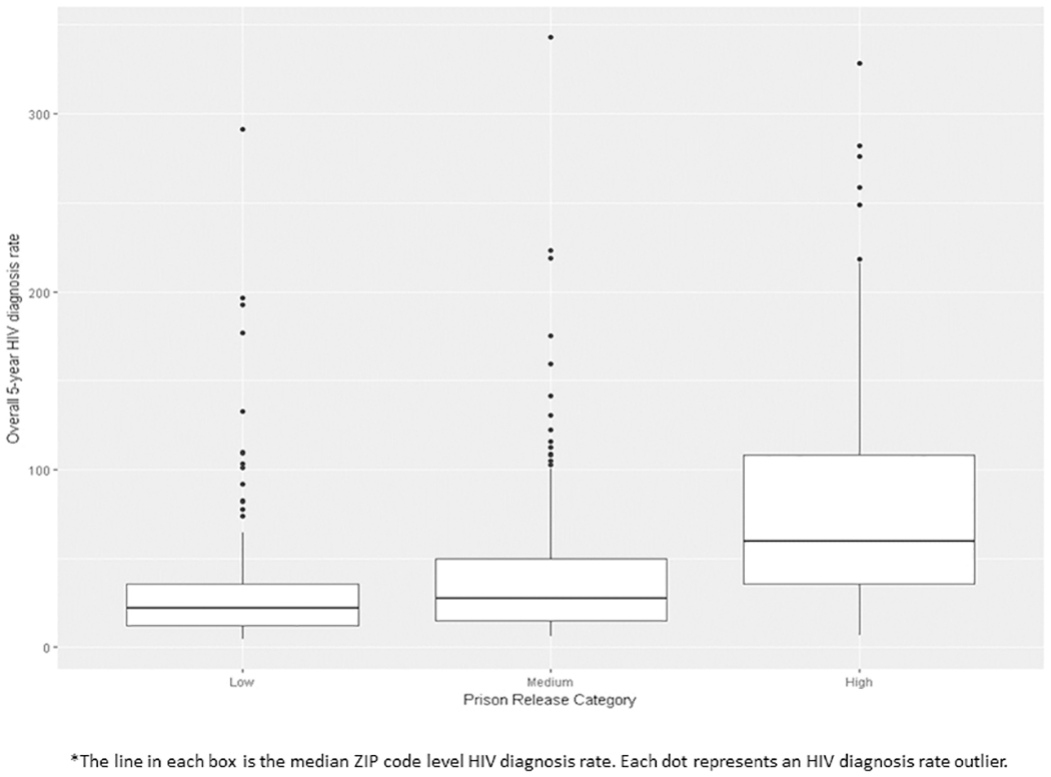
Boxplots of 5-year HIV diagnoses rates by prison release category (low, medium, high)*.

**Fig 3:**
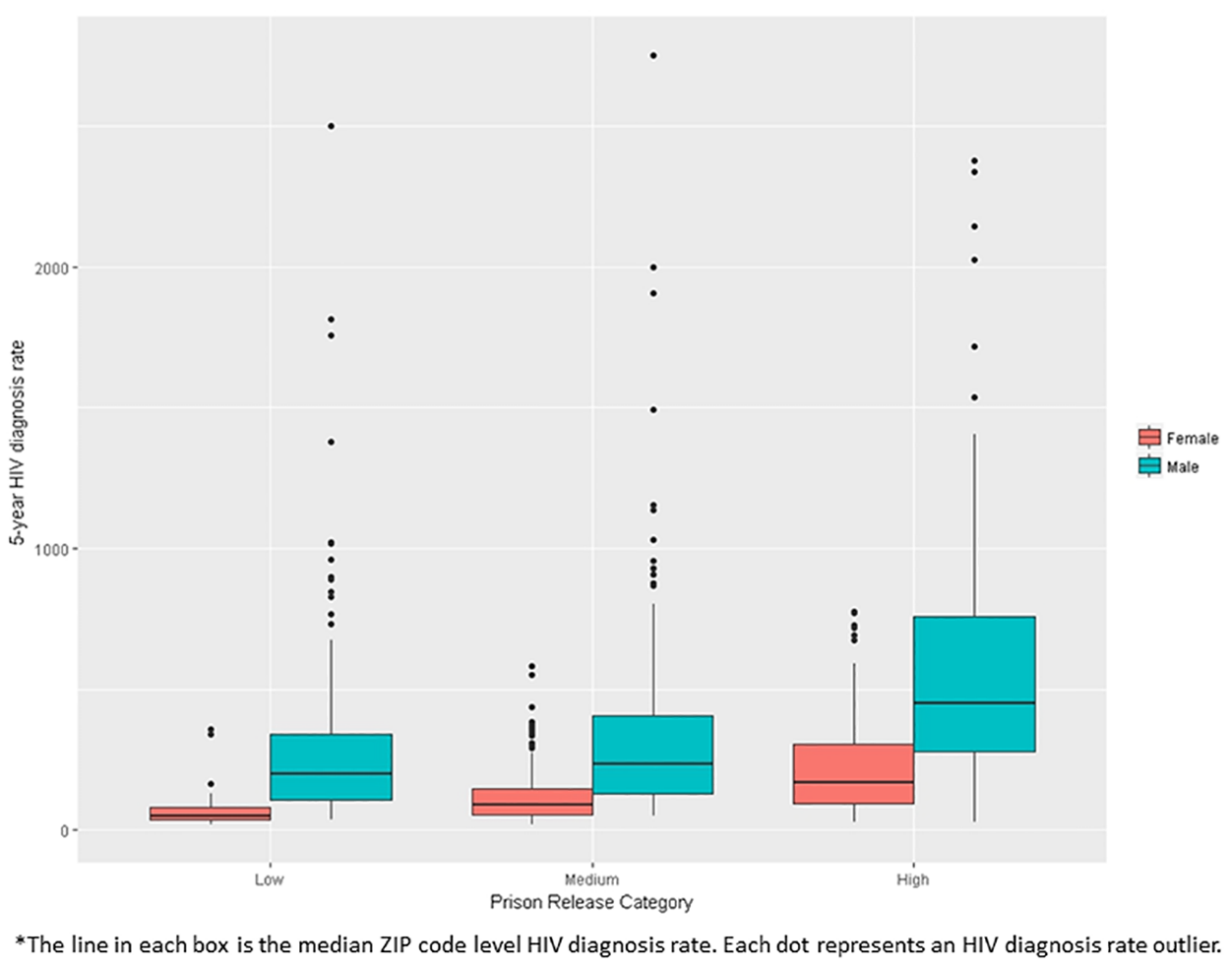
Boxplots of 5-year HIV male and female diagnosis rates by prison release category (low, medium, high)*.

Of 600 ZIP codes included in this analysis, 153 (25.5%) were defined as low, 299 (49.8%) were medium, 148 (24.7%) were defined as having a high prison release rate. For overall, male and female categories, HIV diagnoses rates increased as prison release rate increased. At each prison release level, males had a higher diagnosis rate than females (Figs 2 and 3).

### Regression models

In univariate negative binomial regression models, prison release rate was positively associated with overall 5-year HIV diagnosis rate [Unadjusted Rate Ratio (RR) 1.02 [(95%CI 1.02, 1.03), p<0.001], male 5-year HIV diagnosis rate [RR 1.02 [(95%CI 1.02, 1.03), p<0.001], and female 5-year HIV diagnosis rate [RR 1.06 [(95%CI 1.05, 1.06), p<0.001]. (Table 3)

**Table 3:** Univariate negative binomial regression models of 5-year overall, male and female HIV diagnoses rates.

Variables	Overall 5-year HIV Diagnosis Rate Unadjusted Rate Ratio (95%CI) N = 600	Male 5-year HIV Diagnosis Rate Unadjusted Rate Ratio (95%CI) N = 600	Female 5-year HIV Diagnosis Rate Unadjusted Rate Ratio (95%CI) N = 600
Percent Living in Poverty	1.06*** (1.05, 1.07)	1.05*** (1.05, 1.06)	1.12*** (1.11, 1.13)
Prison Release Rate	1.02*** (1.02, 1.03)	1.02*** (1.02, 1.03)	1.06*** (1.05, 1.06)
GINI	228.57*** (227.51, 229.62)	440.20*** (439.12, 441.28)	1,054.19*** (1,051.74, 1,056.64)
Percentage Non-Hispanic Black Population	1.07*** (1.06, 1.07)	1.06*** (1.05, 1.07)	1.14*** (1.12, 1.15)

***p<0.001

All multivariate regression models included the population-level economic and demographic variables noted to have a positive association with overall HIV diagnosis rate in bivariate analysis (prison release rate, GINI coefficient, percent living in poverty, and percent Non-Hispanic Black population). In the first multilevel model, for overall HIV diagnosis rate, the adjusted rate ratio (ARR) for prison release rate was 1.004 [(95%CI 1.0007, 1.006), p<0.01]. Therefore, a 10 person increase in prison release rate would result in a 4% increase in overall 5-year HIV diagnosis rate—approximately 9.4 additional cases per 100,000 population. A 1 percent increase in percent living in poverty would result in a 2% increase in overall 5-year HIV diagnosis rate—approximately 4.7 cases per 100,000 population [ARR 1.02 (95%CI 1.01, 1.03), p<0.001]. A 0.1 unit increase in GINI coefficient would result in an 18% increase in overall 5-year HIV diagnosis rate—approximately 42.4 additional cases per 100,000 population [ARR 18.0 (95%CI 9.0, 44.7), p<0.001]. A 1 percent increase in percent Non-Hispanic Black population would result in a 5% increase in overall 5-year HIV diagnosis rate—approximately 11.8 additional cases per 100,000 population.

In the second multilevel model, we tested the hypothesis that gender would modify the association of prison release rate on HIV diagnosis rate. The addition of the interaction term (prison release x gender) was noted to result in an ARR of 1.03 [(95%CI 1.02, 1.04), p<0.01]. In gender-stratified, multilevel models, prison release rate was significantly associated with HIV diagnosis rate for males [ARR 1.004 (95%CI 1.0004, 1.007), p<0.01], but not for females [ARR .99 (95%CI 0.99, 1.0), p = NS]. For male HIV diagnosis rate, economic inequality resulted in an ARR of 59.7 [(95%CI 25.0, 144.0), p<0.001], percent Non-Hispanic Black population ARR of 1.05 [(95%CI 1.04, 1.06), p<0.001], and percent living in poverty ARR of 1.02 [(95%CI 1.01, 1.03), p<0.001]. For female HIV diagnosis rate, percent Non-Hispanic Black population resulted in an ARR of 1.09 [(95%CI 1.07, 1.11), p<0.001] and percent living in poverty ARR of 1.06 [(95%CI 1.04, 1.07), p<0.001]. Economic inequality was not found to be significantly associated with female HIV diagnosis rate. (Table 4)

**Table 4:** Multilevel regression models of overall and gender stratified 5-year HIV diagnoses rates.

	Overall 5-year HIV Diagnosis Rate	Overall 5-year HIV Diagnosis Rate w/Effect Modification	Gender Stratification	
			Male 5-year HIV Diagnosis Rate	Female 5-year HIV Diagnosis Rate
	Adjusted Rate Ratio(95%CI) N = 600	Adjusted Rate Ratio(95% CI) N = 1200	Adjusted Rate Ratio(95%CI) N = 600	Adjusted Rate Ratio (95%CI) N = 600
Variables				
Percent Living in Poverty	1.02***(1.01, 1.03)	1.00 (0.99, 1.00)	1.02*** (1.01, 1.03)	1.06***(1.04, 1.07)
Prison Release Rate	1.004**(1.0007, 1.006)	---	1.004** (1.0004, 1.007)	.99 (0.99, 1.00)
Prison Release Rate x Gender	---	1.03**(1.02, 1.04)	----	---
GINI	18.0 ***(9.0, 44.7)	42.0**(31.0, 53.0)	59.7*** (25, 144)	1.2 (0.2, 7.6)
Percentage Population (Black)	1.05***(1.04, 1.06)	1.04**(1.03, 1.05)	1.05***(1.04, 1.06)	1.09***(1.07, 1.11)
Log likelihood	-4720.1	-4492.9	-2514.8	-1659.3
AIC	9456.2	9005.7	5045.7	3334.7
BIC	9496.8	9056.6	5080.8	3369.8

*p<0.05,

**p<0.01,

***p<0.001

## Discussion

This study investigated the association between prison release and HIV diagnosis rate in 9 cities in the southern region of the US. On a population-level, we confirmed findings from previous ecological studies and found that HIV diagnosis rate is most consistently and strongly correlated with economic (poverty and inequality) and demographic (race) characteristics [17,18]. However, our study found that prison release is an important population-level factor with significant association with neighborhood-level HIV diagnosis rate in the South.

Prison release is a critical period during which incarcerated individuals living with HIV must be linked with community-based care and treatment. The Centers for Disease Control and Prevention (CDC) has established best practices for discharge planning for incarcerated PLWH which include confidential opt-out testing while incarcerated, linkage to treatment and provision of antiretroviral therapy [28]. However, studies have noted challenges in implementing opt-out testing and comprehensive discharge planning for incarcerated PLWH. In a survey of correctional facilities, Solomon et al found that less than 20 percent of prison systems provide opt-out HIV testing and only 19 percent provided discharge services that met the CDC’s criteria [13]. In two separate analyses, Baillargeon et al found that only 30 percent of PLWH filled a prescription for antiretroviral therapy within sixty days of discharge and only 28 percent were enrolled in clinical care within 90 days [29,30]. Lack of connection to clinical care not only compromises individual health, but increases the risk of onward HIV transmission.

In addition to the direct impact that poor linkage to treatment may have on onward HIV transmission, incarceration indirectly reduces the economic opportunities of released individuals. Former inmates experience high rates of unemployment. Once employed they earn less per hour and reap lower annual earnings than individuals without a history of incarceration. Unemployed or underemployed PLWH who are released have a high likelihood of re-engagement in risk behaviors that promote onward HIV transmission, such as transactional sex and drug use. In addition, removing adults who are the primary breadwinner from their homes disrupts family relationships, damages social networks, and perpetuates a cycle of poverty [31].

Improving discharge planning and the economic opportunities for released inmates is a timely concern. To address the massive cost of incarceration, criminal justice reform and early release for non-violent offenders has been implemented [32]. Therefore, more individuals, including PLWH, will require these services. Several studies have demonstrated that discharge planning prior to release improves clinical outcomes [30,33]. Correctional facilities within cities where early release is occurring should provide a comprehensive array of services locally to meet the needs of PLWH.

In this study we hypothesized that gender would modify the association of prison release rate on overall HIV diagnosis rate. Males are incarcerated at a higher rate than females, and HIV diagnosis rate is higher among men than among women. In gender stratified models prison release was significantly associated with male HIV diagnosis rate. No association was noted for female HIV diagnosis rate. The most likely reason for this finding is that onward HIV transmission in the U.S. is most frequently due to male same sex behavior. Men are also more likely to engage in substance use, including injection drug use which increases risk for HIV transmission [34]. Analyses should be undertaken to determine whether differences in this association are noted by gender within specific cities.

Our findings suggest a stronger association between prison release and HIV diagnosis rate in certain cities. The strongest association was noted in Baton Rouge, LA which may indicate specific challenges within that jurisdiction or the entire state. Louisiana has an incarceration rate that is 102% higher than the national average and is the highest nationwide [35]. A recent report noted significant problems in implementing HIV testing programs, providing HIV treatment, and planning discharges in Louisiana [36]. Additional research is needed to understand the characteristics promoting the associations noted within each city in order to develop contextually appropriate interventions.

This study has several limitations. The primary unit of analysis is the ZIP code rather than the individual which is a likely source of ecological fallacy. We cannot determine if incarcerated individuals are more likely to transmit HIV post release. However, our conceptual model presents a hypothesis of how many individual and community-level factors may impact HIV incidence. ZIP codes are large areas that usually encompass several neighborhoods. Therefore, characteristics ascribed to one ZIP code may not be consistent within all parts of that ZIP code. If data were available, studies should explore individual and population level data in tandem to fully understand the role of exposures on HIV diagnosis rate. It is important to note that the discharge address data is self-reported and not confirmed by independent sources [21]. In addition, temporal association between the prison release data and the HIV diagnoses rate data are approximate. We used prison release data from 2008 and HIV diagnoses data aggregated from 2010 through 2014 because these data were publicly available. Lastly, we assessed the association of prison release rate and HIV diagnosis rate, not discharge from other correctional facilities (i.e. jails). Ideally, an analysis such as this would include jails because the turnover rate is higher than in prisons due to the shorter median stays.

Opportunities to decrease the risk of onward HIV transmission from correctional facilities to home communities exist while incarcerated and at discharge. Release from prison is a particularly vulnerable period where PLWH risk non-adherence to treatment. Previous studies have noted the detrimental impact that prison release without adequate discharge planning has on clinical outcomes. This study advances the literature by exploring the role of prison release on the HIV epidemic beyond the individual to the community. Future studies are needed to understand the impact of prison release within specific cities to develop interventions to decrease the impact of mass incarceration on HIV diagnoses throughout the US.
